# Characterization of transposable elements within the *Bemisia tabaci* species complex

**DOI:** 10.1186/s13100-022-00270-6

**Published:** 2022-04-19

**Authors:** Juan Paolo A. Sicat, Paul Visendi, Steven O. Sewe, Sophie Bouvaine, Susan E. Seal

**Affiliations:** 1 Natural Resources Institute, University of Greenwich, Central Avenue, Gillingham, Chatham ME4 4TB UK; 2 Centre for Agriculture and the Bioeconomy, Queensland University of Technology, Brisbane, QLD 4000 Australia

**Keywords:** Transposable elements, Whitefly, Bioinformatics, *Bemisia tabaci*, DNA transposons, TE annotation

## Abstract

**Background:**

Whiteflies are agricultural pests that cause negative impacts globally to crop yields resulting at times in severe economic losses and food insecurity. The *Bemisia tabaci* whitefly species complex is the most damaging in terms of its broad crop host range and its ability to serve as vector for over 400 plant viruses. Genomes of whiteflies belonging to this species complex have provided valuable genomic data; however, transposable elements (TEs) within these genomes remain unexplored. This study provides the first accurate characterization of TE content within the *B. tabaci* species complex.

**Results:**

This study identified that an average of 40.61% of the genomes of three whitefly species (MEAM1, MEDQ, and SSA-ECA) consists of TEs. The majority of the TEs identified were DNA transposons (22.85% average) while SINEs (0.14% average) were the least represented. This study also compared the TE content of the three whitefly genomes with three other hemipteran genomes and found significantly more DNA transposons and less LINEs in the whitefly genomes. A total of 63 TE superfamilies were identified to be present across the three whitefly species (39 DNA transposons, six LTR, 16 LINE, and two SINE). The sequences of the identified TEs were clustered which generated 5766 TE clusters. A total of 2707 clusters were identified as uniquely found within the whitefly genomes while none of the generated clusters were from both whitefly and non-whitefly TE sequences.

This study is the first to characterize TEs found within different *B. tabaci* species and has created a standardized annotation workflow that could be used to analyze future whitefly genomes.

**Conclusion:**

This study is the first to characterize the landscape of TEs within the *B. tabaci* whitefly species complex. The characterization of these elements within the three whitefly genomes shows that TEs occupy significant portions of *B. tabaci* genomes, with DNA transposons representing the vast majority. This study also identified TE superfamilies and clusters of TE sequences of potential interest, providing essential information, and a framework for future TE studies within this species complex.

**Supplementary Information:**

The online version contains supplementary material available at 10.1186/s13100-022-00270-6.

## Introduction

Whiteflies are agricultural pests that cause crop losses amounting to billions of dollars[1–3]. More than 1500 whitefly species have been identified and amongst them, the members of *Bemisia tabaci* whitefly species complex are the most damaging collectively in terms of their broad crop host range (e.g. beans, cassava, cotton, potato, tomato) and ability to serve as a vector for > 400 plant viruses [4–6].

Agricultural intensification and climate change have led to highly fecund populations of *B. tabaci* spreading across continents and globally through international trade of infested plants [1, 7, 8]. The severity of this pest species complex has for decades shaped several national and international collaborative projects [3], with a dramatic increase in genome and transcriptome resources in the past decade. These have assisted in the exploration of mechanisms that underly diversification within this pest species complex, such as differing host specificities and detoxification mechanisms, and plant virus interactions [9–13]. In the last few years, draft genome assemblies have been published (MEAM1, MED/Q, and SSA-ECA) alongside the annotation of genomic features that are associated with insecticide resistance, detoxification, and virus transmission [14–16]. Transposable elements (TEs) have, however, been neglected in all of these studies with no detailed characterization to date of TEs found in this whitefly species complex.

The identification of TEs is integral in the analysis of genome assemblies as TEs are abundant in eukaryotic genomes and can multiply, move, affect gene regulation, and expand the host’s genome [17–21]. TEs are classified into two main classes based on their method of transposition: DNA transposons and Retrotransposons [22–25]. DNA transposons transpose with the aid of a DNA intermediate and can either be autonomous or non-autonomous [23, 26, 27]. Autonomous elements can transpose on their own while the non-autonomous elements require other TEs to facilitate their movement [27, 28]. The majority of DNA transposons utilize a “cut-and-paste” method of transposition; wherein the transposons are “cut” from their position and then “pasted” (inserted) into a new target site [18, 28, 29].

Retrotransposons are TEs that can transpose with the aid of an RNA intermediate [30–32]. While DNA transposons encode for a transposase, retrotransposons produce RNA transcripts, and they are transcribed from RNA to DNA with the aid of reverse transcriptase enzymes and the sequence is then integrated into new sites in the genome [30, 31]. Their mobilization in the genome does not require excision hence their movement has been dubbed as “copy-and-paste” [31, 33]. Retroelements can be further classified based on their structures into two orders: Long terminal repeat (LTR) retrotransposons and NonLTR retrotransposons [30–32].

TEs can be further classified into superfamilies and their presence in the different arthropod species varies greatly, currently ranging from as low as 2.6% in *Belgica antartica* to as high as 72.8% in *Sitophilus oryzae* [34, 35]. The functions of these elements are often unknown, but their presence in genomes has been associated with inducing various changes in their host organism. The majority of TE studies in insects have been in drosophilids and one of the most characterized TE is the P element [36, 37]. P elements were first discovered in *Drosophila melanogaster* and were shown to cause hybrid dysgenesis [38], which occurs when female strains of *D. melanogaster* that lack P elements mate with male strains with autonomous P elements [36, 39]. The resulting combination results in progeny with sterility disorders, an elevated mutation rate, and increases in chromosomal rearrangements and recombination [36, 39, 40]. Different types of TEs have different effects and the characterization of these elements in other insect species has underpinned an improvement in our understanding of the potential impacts of these elements.

The roles that TEs can play in gene regulation and expression have already been described [28, 41–46] and the abundance and types of TEs in the different whitefly genomes could have shaped the evolution of the species complex. TEs have also been associated with gene duplication wherein the insertion location of the TE affects the normal replication process [17, 47]. The exact mechanism of the alteration of the process depends on the type of TE and the extent of its effects vary accordingly [44, 47–49].

TEs represent a major proportion of *B. tabaci* genomes, accounting for approximately 40–44% of the published draft genomes of two *B. tabaci* species termed Middle East Asia Minor 1 (MEAM1) and Mediterranean Q (MED/Q) [14, 16]. The latest released *B. tabaci* draft genome of a SubSaharan African population (SSA-ECA), reported a slightly lower (38.5%) TE content but it was noted that the 513 Mb genome assembly was missing around a quarter of genome data [15]. Hence the repeat content of the SSA-ECA genome cannot be considered as accurate.

Aside from the proportion of TEs found within the *B. tabaci* genomes, little is known on the TEs found within the *B. tabaci* species complex. In addition, there are marked differences in reported estimates of TE orders between the above two complete *B. tabaci* draft genomes. Although all the studies reported around ~ 40% of the genomes to be comprised of TEs, the MEAM1 and SSA-ECA whitefly genomes were reported to have an abundance of DNA transposons [14, 15] particularly MITEs (miniature inverted-repeat transposable elements) while LTRs were reported [16] to be the most abundant in the MED/Q genome. Members of the *B. tabaci* species complex show very different biological and phenotypic properties and hence these contrasting results were considered potentially significant.

The studies that reported very different TE class proportions in the *B. tabaci* whitefly genomes [14–16] employed different TE annotation workflows. In both MEAM1 and SSA-ECA annotation [14, 15], a MITE-specific identification tool was included (MITE-Hunter), whereas LTR-specific identification tool (LTR-Finder) was incorporated in the MED/Q repeat annotation workflow [16]. Chen et al. [14, 15] created their species-specific repeat libraries using RepeatModeler (RECON and RepeatScout) and included MITE-Hunter for the identification of MITEs. Xie et al. [16] used Piler-DF, and RepeatScout to create their repeat library and included LTR-FINDER to identify LTRs.

The three whitefly draft genome assemblies used different genome sequencing technologies and assembly methods. MEAM1 whitefly DNA was sequenced using Illumina HiSeq 2500 system, and Illumina paired end reads assembled by Platanus v1.2.1, with gaps subsequently filled using PacBio long reads and PBJelly [14]. The MED/Q genome assembly was also constructed from Illumina paired end reads, but assembled using SOAPdenovo [16] followed by using Bacterial Artificial Chromosome (BAC) libraries to improve assemblies. For the most recently released SSA-ECA draft genome assembly, paired end and mate pair libraries from HiSeq 2500 were used with Platanus [15]. Pilon was included to fill in gaps. The SSA-ECA publication [16] noted that although ~ 25% of the genome was missing, the majority of the gene space was considered to have been assembled correctly.

The use of different assembly methods and workflows hinders the accurate comparisons of TE classes previously reported across the three *B. tabaci* genomes. Reliable inferences based on the significant differences in TE compositions found across the published genomes of the *B. tabaci* species complex can therefore not be made. Furthermore, attempts were made to replicate the identification workflows reported in the published data and results were inconsistent with the published estimates using the same genome assemblies. To address the issue of the assemblies using different TE annotation workflows, this study developed a reproducible workflow for identifying and classifying TEs found within *B. tabaci* genomes. The application of the same workflow across all the published *B. tabaci* genomes provided a standardized TE annotation process and highlighted some misclassification and an overestimate of TE compositions in currently published *B. tabaci* genomes. This study provides the first accurate exploration of TE classes in the *B. tabaci* species complex.

## Results

### Identification of TEs using the repeatmasker repBase library

The three draft genomes (MEAM1, MED/Q, and SSA-ECA) for the *B. tabaci* cryptic species complex published to date were the focus of analyses. TEs within these genomes were initially identified using a RepBase library (version RepBase_RepeatMasker-edition20180826 library) through RepeatMasker. The results of the TE identification using the RepeatMasker RepBase library were significantly lower than reported in their respective publications (Table 1); MEAM1 (18.92% vs 43.82% published), MED/Q (17.28% vs 40.29% published), and SSA-ECA (13.41% vs 38.52% published).

**Table 1:** Repetitive elements identified in the three whitefly genomes

	**MEAM1**			**MED/Q**			**SSA-ECA**		
	**Published**	**RepBase**	**Custom Library**	**Published**	**RepBase**	**Custom Library**	**Published**	**RepBase**	**Custom Library**
**DNA**	29.25	18.07	25.28	15.66	16.48	23.42	25.94	12.92	19.86
**Retroelements**		0.86	2.6		0.61	2.65		0.42	1.72
**LINE**	0.96	0.61	1.25	3.18	0.57	0.96	0.44	0.38	0.94
**SINE**	0.16	0.04	0.17	0.96	0.04	0.18	0.16	0.04	0.08
**LTR**	0.49	0.21	1.19	18.5	0.19	1.51	0.08	0.07	0.7
**Unknown**	12.96	0	16.26	1.99	0	14.81	11.9	0	15.22
**Total**	43.82	18.92	44.14	40.29	17.28	40.88	38.52	13.41	36.8

Results of the identification of TEs reported by their respected studies, using the last publicly available RepBase library (RepBase RepeatMasker-edition20180826), and the custom-built repeat library built using the workflow described in the study

The RepBase library was searched for *B. tabaci*-specific TEs and 282 different TE consensus sequences were identified. The result of the identification showed that only some of the identified TE consensus sequences were submitted to RepBase and with these submitted consensus TE sequences, only less than half of the published TEs were identified. Attempts to find the rest of the consensus sequences in publicly available repositories were unsuccessful.

The RepBase library was therefore tested for its ability to identify TEs in a *Drosophila melanogaster* genome (release 6 [50]) to identify if the anomalies for the hemipteran genomes tested in this study were due to user errors. The RepBase library was able to identify 17.44% TE genome proportion while published results show that < 20% of the genome was identified as TEs in different Drosophila studies [51–54]. The results of the identification were thus in line with what was reported to be found in the species, confirming that the library was being searched correctly.

The results of the TE identification using the RepeatMasker RepBase library showed that the library could not be used for the characterization and comparison of the TEs found within the whitefly genomes. To resolve the issue, an annotation workflow was developed to standardize the identification of the TEs across the whitefly genomes. This had varied in the published research that utilized different TE identification tools; MEAM1, and SSA-ECA [14, 15] used a DNA transposons specific tool, while MED/Q [16] used a LTR specific identification tool. Standardization of the annotation workflow is required for an accurate comparison of TEs across the three genomes. A species-specific custom-built repeat library was created for each genome studied using the same tools to identify and classify TEs within each genome. The identification of the TEs in the workflow combines several methods in the identification of elements: structural-based and de novo; while the classification of the identified elements uses sequence similarity, structural, and machine learning (for details see Methodology section).

The performance of the annotation workflow developed was validated using a well characterized genome to determine its suitability for annotating TEs in less well characterized insect genomes. The *D. melanogaster* genome (release 6 [50]) was chosen for the validation as it is known to be one of the most accurate in terms of its TE annotation with several iterations of reference genome releases and information on TEs released alongside these [50, 55]. The annotation workflow developed was compared against the RepeatMasker RepBase library as the latter uses a database that contains the updates from several TE studies and libraries that includes the TE annotation from the *D. melanogaster* genome releases [24, 56].

A total of 17.44% genome proportion of interspersed repeats was found in the *D. melanogaster* using the RepeatMasker library compared to 16.88% genome proportion of interspersed repeats was found using the species-specific custom-built library (Table 2). Most of the repeats found were LTRs and a difference of 0.46% in this category was seen between the RepeatMasker and custom-built libraries. The SINE class of elements was the least common; the RepeatMasker library identified 81 bp of SINEs while the custom-built library found none (0 bp). For DNA transposons a difference of 0.58% was observed between the two libraries, while a difference of 0.42% was observed in the detection of LINEs. The difference of < 1% of the total of TEs identified in the *D. melanogaster* genome and less than < 1% in each of the orders support the capability of the workflow developed in identifying TEs found within a genome.

**Table 2:** RepeatMasker output of RepeatMasker library and the species-specific custom-built library for the Drosophila melanogaster genome (release 6 [50])

	**RepBase (%)**	**Custom Library (%)**
DNA	1.79	1.21
LINE	4.93	4.50
SINE	< 0.001	0.00
LTR	10.68	10.22
Unclassified	0.04	0.34
Total Interspersed Repeats	17.44	16.88

Comparison of the results of the identification of TEs using RepeatMasker RepBase library and the species-specific repeat library in the *D. melanogaster* genome. The custom-built repeat library was built using the workflow described in the study

### TEs in arthropod genomes

**Fig. 1:**
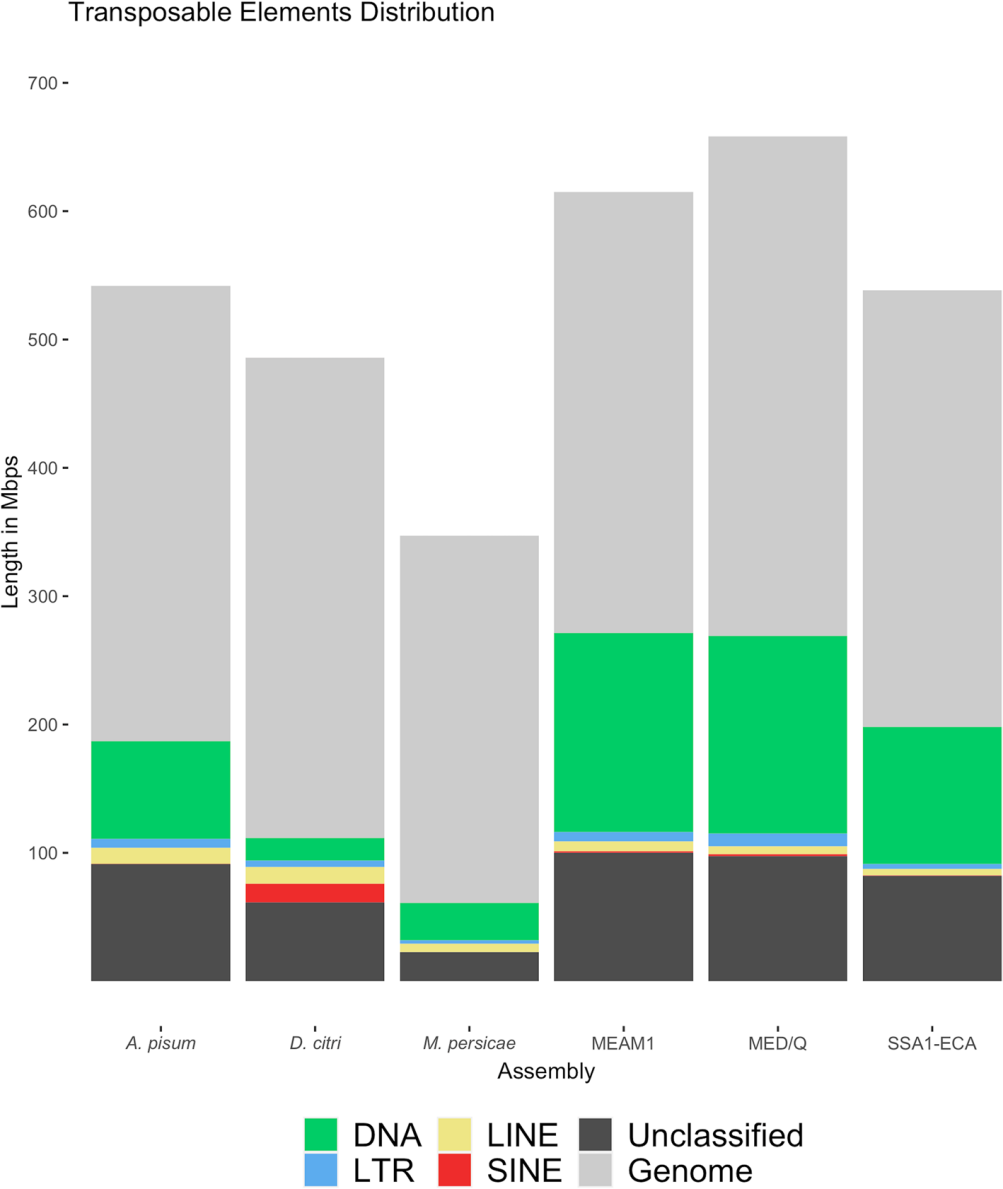
Distribution of transposable elements in each respective genome. Stacked bar chart illustrating the length of each genome and the length occupied by the TEs in each genome. RepeatMasker with the species-specific repeat library was used to identify the TE content of each genome

The validated developed workflow was used to identify the TE content of each of the target genomes (Fig. 1), resulting in a custom-built species-specific library for each of the genomes studied. Aside from the three whitefly genomes (MEAM1, MED/Q, and SSA-ECA), three further hemipteran genomes were included as a general comparison, namely *Acyrthosiphon pisum*, *Diaphorina citri,* and *Myzus persicae*. The three whitefly genomes all had a higher TE content (an average of 40.61% genome proportion of TEs) compared to each of the three non-whitefly genomes (an average of 25.01% TE genome proportion). MEAM1 had the highest TE content across the six genomes at 44.14% while the *A. pisum* assembly had the highest TE content amongst the non-whitefly genomes at 34.54%. The SSA-ECA draft genome (known to be missing ~25% genome data) had the lowest TE content amongst the whitefly genomes at 36.80%, over 2% higher than the TE content in the *A. pisum* genome assembly. The *M. persicae genome assembly* had the lowest TE content across the six genomes at 17.52%.

**Fig. 2:**
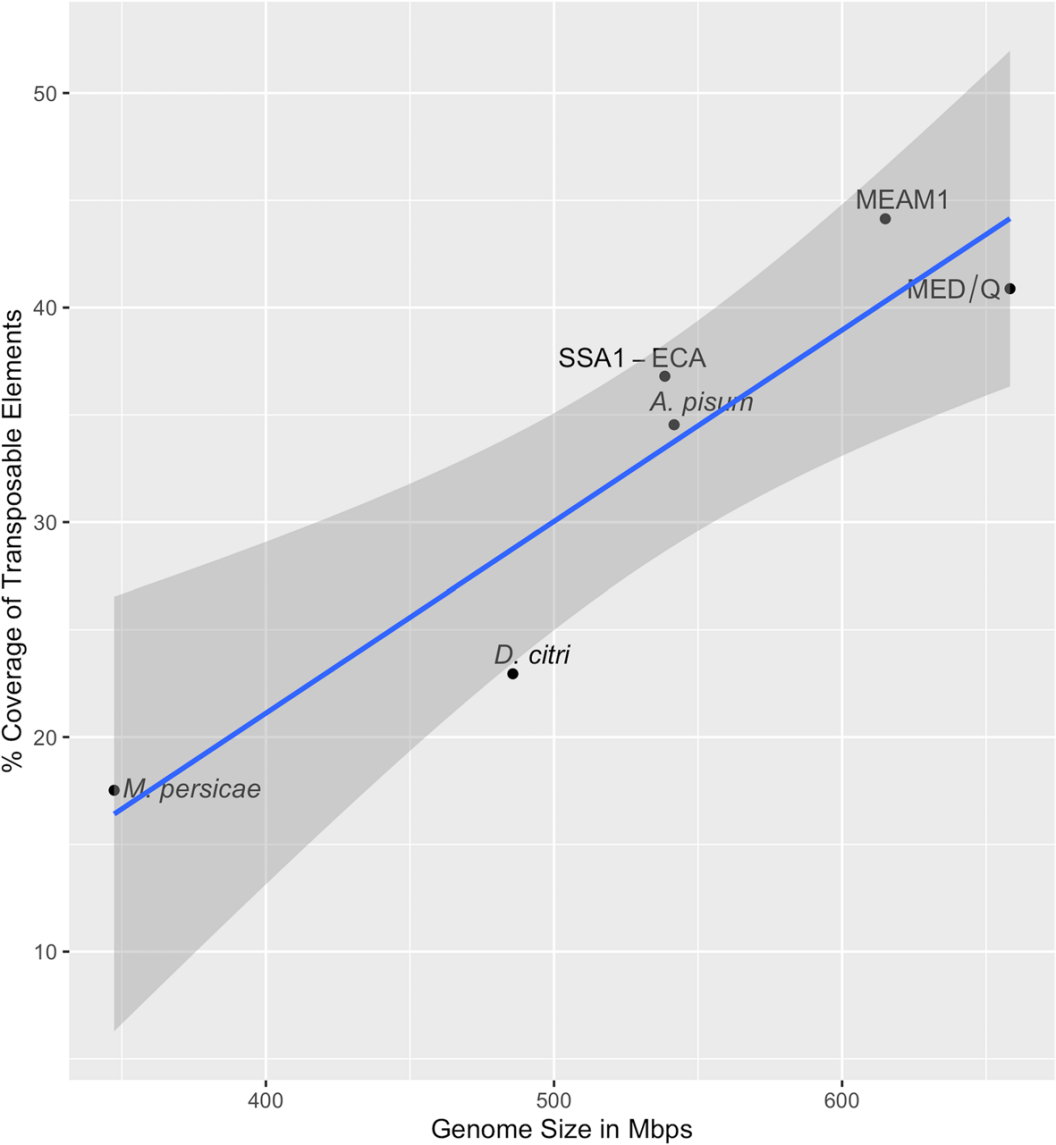
Percent proportion of transposable elements and assembly size. Each genome was plotted in relation to their TE proportion and assembly size. TE proportion in the six genomes is positively correlated with the size of the genome assembly (*p* = 0.006). The grey shaded area represents the 95% confidence interval while the blue line is the regression line (*r* = 0.0.93)

The relationship between assembly sizes of the six genomes and their TE content was tested using Spearman’s rank rho correlation (Fig. 2). TE proportion was found to be positively correlated with assembly size (*r* = 0.93, *p* = 0.006). The highest TE content (44.14%) across the six genomes was in the MEAM1 genome (615 Mbp) while the smallest genome, the *M. persicae* genome assembly (347 Mbp) had the lowest TE content at 17.52%. Amongst the whitefly genomes, SSA-ECA has the smallest assembly size (538.48 Mbp) and the lowest TE genome proportion (36.80%).

### Difference in the distribution of TE content between genomes

There was no statistically significant difference (*p* = 0.09) in genome assembly size between the whitefly genomes (average 603.92 Mbp) and the non-whitefly genomes (average 458.24 Mbp). This allowed us to compare the two groups without significantly biasing our results with the variations in genome assembly sizes. The distribution of TEs as a percentage of genome was compared across the six genomes. The majority of the classified elements within the whitefly genomes were DNA transposons at an average of 22.85% across the three genomes. MEAM1 had the highest distribution amongst the three whitefly genomes at 25.28% while SSA-ECA had the lowest at 19.86%. Retrotransposons were classified at a much lower average of 2.32% proportion in the whitefly genomes, with LTRs as the most abundant order identified across the three at an average of 1.13% followed by LINEs at an average of 1.05%.

For the three non-whitefly genomes, DNA transposons were the most abundant in the *A. pisum* (14.06%) and *M. persicae* (8.35%) genomes while retrotransposons were the most abundant class in the *D. citri* genome (6.68%). An average of 4.34% proportion was identified as retrotransposons within the non-whitefly genomes. LINEs were the most abundant retrotransposon order in the *A. pisum* genome(2.32%) and *M. persicae* genome (1.86%) while SINEs were the most abundant in *D. citri* genome (3%).

Across the four orders of TEs, SINEs were the least identified at an average of 0.58% (0.14% for the whitefly genomes and 1.01% for the non-whitefly genomes). Amongst all the six genomes, the *D. citri* genome had the highest percentage of SINEs at 3% while this TE order was not detected in the *M. persicae* genome assembly.

**Fig. 3:**
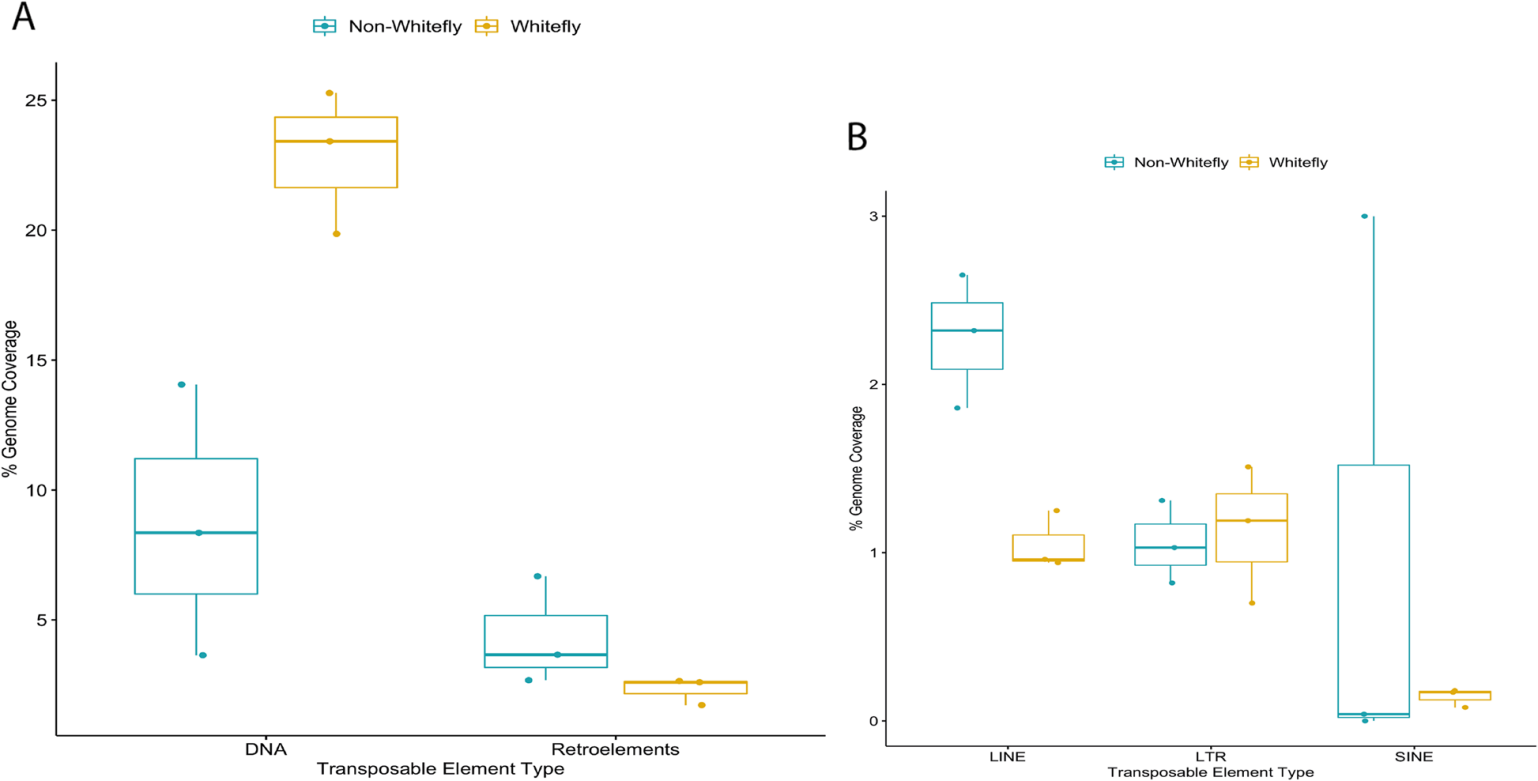
Percent proportion of each order of transposable elements in the non-whitefly and whitefly group. Box plots comparing percent genome proportion of each order of TEs between the non-whitefly and the whitefly group. The box represents the interquartile range (25th to 75th percentile) values and the line in the middle of the box represents the middle quartile (50th percentile or median). The upper whisker represents the values 1.5 times larger than the 75th percentile and the lower whisker represents the values smaller than the 25th percentile. (A) The overview of the distribution of TEs classes between the non-whitefly and whitefly groups. The majority of the TEs identified were DNA transposons, most abundant in whitefly genomes. (B) The comparison of the distribution of retrotransposons between the non-whitefly and whitefly group. SINEs distribution varies significantly across the non-whitefly group; the *D. citri* genome assembly having the highest at 3% while none was detected in *M. persicae genome assembly*

The distribution of TEs between the genomes was explored further by comparing their distribution between the two groups of genomes to determine if there were any specific features that appeared to be specific to the whitefly genomes studied. The comparison of the distribution of the orders of the TEs between the whitefly and the non-whitefly genomes was performed using a two-sample t-test (DNA transposon, LTR, and LINE) and Wilcoxon rank-sum test (SINE) (Fig. 3). A standard t-test was used for orders that had the same variance (DNA transposons, LTRs, and LINEs) while a Wilcoxon rank-sum test for SINEs as the distribution for genome proportion in the two groups as they had a non-normal distribution. There is a significant difference between the mean TE content of DNA transposons (*p* = 0.01) and LINEs (*p* = 0.008) between the whitefly genomes and the non-whitefly genomes, while there was no significant difference found in LTRs (*p* = 0.7856) and SINEs (*p* = 0.6625). There are significantly more DNA transposons found in the whitefly genomes and significantly less LINEs compared to the three non-whitefly hemipteran genomes studied.

Unclassified elements are still found within the identified TEs. Across the six genomes, an average of 13.70% genome proportion remains unclassified (15.43% for the whitefly genomes and 11.98% for the non-whitefly genomes). The unknown consensus sequences from the whitefly species-specific TE libraries were searched against the NCBI non-redundant protein database and UniProtKB/Swiss-Prot Arthropoda protein sequences. The repeat sequences with hits were planned to be excluded from the final TE library; however, no matches were found.

Lastly, it should be noted that the relative proportions of the elements will be subject to change when the unclassified elements become classified; nevertheless, the very high proportion of identified DNA transposons in the whitefly genomes means that this class will remain the largest order of elements identified within all three whitefly genomes analyzed (Supplementary Table 2).

### TE superfamilies across the genomes

Each TE from the different orders can be further classified into superfamilies on the basis of their monophyletic origin and homology of motifs [27, 56, 57]. Superfamilies were identified in each genome (Table 3). A total of 98 TE superfamilies were identified in the whitefly genomes and 89 for the non-whitefly genomes. A total of 69 TE superfamilies were identified to be present across the genomes in the two groups (39 DNA transposon, eight LTR, 19 LINE, and three SINE). Most of the superfamilies identified were classified as DNA transposons with a total of 66 different superfamilies of which 19 were unique to whitefly genomes while eight were unique to non-whitefly genomes. SINE superfamilies were the least identified with 11 superfamilies of which four are unique to whitefly genomes and another four unique to the non-whitefly genomes. LINE superfamilies were the most identified retrotransposons with 29 unique superfamilies of which three are unique to whitefly genomes while seven are unique to the non-whitefly genomes.

**Table 3:** Repeat Superfamilies identified within the genomes

	**DNA**	**LINE**	**LTR**	**SINE**	**Total**
MEAM1	48	20	9	5	82
MED/Q	49	20	6	4	79
SSA-ECA	44	18	9	4	75
*A. pisum*	43	18	5	1	67
*D. citri*	30	23	6	7	66
*M. periscae*	36	18	7	0	61

The table presents a summary of the number of superfamilies found in each class of TEs in each of the genomes. DNA represent DNA transposons, *LINE* Long interspersed nuclear elements, *SINE* Short interspersed nuclear elements, *LTR* Long terminal repeats

MEAM1 showed the greatest number of superfamilies identified at 82 while the *M. persicae* genome had the lowest at 61 superfamilies. In all genomes, DNA transposon superfamilies were the most identified with an average of 47 in the whitefly genomes and 36 in the non-whitefly genomes. MED/Q and MEAM1 had the greatest number of DNA transposon superfamilies at 49 and 48 respectively, while the *D. citri* genome had the least at 30 superfamilies. SINE superfamilies were the least identified at an average of four superfamilies. The *D. citri* genome had the greatest number of SINE superfamilies identified with seven while SINEs were not identified at all in *M. persicae* genome assembly.

Further analysis of the superfamilies found across the genome assemblies was performed by clustering the TE consensus from the six species-specific libraries. The clustering was based on the length of the TEs and 80% sequence similarity. A total of 5766 clusters were created; 1131 clusters from the non-whitefly TE consensus sequences, 2707 clusters from whitefly TE consensus sequences, and 1928 clusters created from TE consensus sequences found in the same genome assembly (Supplementary Table 3). The 1928 clusters from TE consensus sequences found in the same genome assembly were expected. These clusters were created from the same superfamilies found within the species-specific library of one genome (i.e. Gypsy element from MEAM1 identified as 80% similar to another MEAM1 Gypsy element). These expected overlaps are disregarded as clustering was already performed during the creation of the species-specific TE libraries (see Methodology). Although similar repeat superfamilies were identified across the genome assemblies based on their classification order, none of the TE consensus sequences from each group (whitefly vs. non-whitefly) was identified as shared based on their sequence similarity and length.

Breakdown of the 2707 clusters from the whitefly TEs (Table 4) reveals that MEAM1 and MED/Q genomes shared the greatest number of clusters at 987 while MED/Q and SSA-ECA shared the least at 441. A total of 733 clusters were identified as shared across the three *B. tabaci* genomes. There were 216 known TE clusters identified as DNA transposons of which 37 clusters were from Helitrons, 31 clusters were from different hAT families, and 31 were from different TcMar families. A total of 174 clusters are identified as LTRs and three superfamilies account for most of these clusters: 61 Copia clusters, 56 Gypsy clusters, and 54 Pao clusters. A total of 120 clusters are identified as LINEs and three superfamilies account for more than half of the clusters: 25 Jockey clusters, 19 L2 clusters, and 17 R1 clusters. Clusters classified as SINEs were identified the least, with only 2 SINEs clusters from the 733 clusters.

**Table 4:** Number of clusters shared across the three *B. tabaci* genomes

	**DNA**	**LTR**	**LINE**	**SINE**	**Unknown**	**Retroelements**	**Total**
ALL	216	174	120	2	220	1	733
MEAM1 and MED/Q	273	212	179	4	318	1	987
MEAM1 and SSA-ECA	133	74	56	0	283	0	546
MED/Q and SSA-ECA	126	74	56	0	183	2	441
Total	748	534	411	6	1004	4	2707

The table presents a summary of the number of clusters identified as shared across the *B. tabaci* genomes*. *The clusters were created from the TE consensus sequences from the six genomes included in the study. DNA represent DNA transposons, *LINE* Long interspersed nuclear elements, *SINE* Short interspersed nuclear elements, *LTR* Long terminal repeats

Lastly, a significant number (1004) of clusters from the whitefly TE clusters were from unclassified TE consensus sequences. Unclassified TE consensus sequences from MEAM1 and MED/Q created the greatest number of clusters at 318 clusters, followed by TEs from MEAM and SSA-ECA at 283 clusters and a total of 220 clusters were created from the three whitefly TE consensus sequences.

### Repeat landscapes

With the help of a script included in RepeatMasker, several repeat landscapes were produced. These repeat landscapes show the sequence divergence measured by Kimura distance within each genome. The graphs below present the distribution of genome coverage of copies of each type of transposable element (DNA transposon, LTR, LINE, SINEs, Unknown, and others) and its divergence from their consensus sequence. A copy’s divergence can infer its activity and age of insertion. A low divergence score implies a more recent transposable element activity while more divergent scores represent copies with older transposition events. Peaks of activity can also be observed in these graphs, and they represent transposition bursts in the evolutionary history of the specific transposable elements [58].

**Fig. 4:**
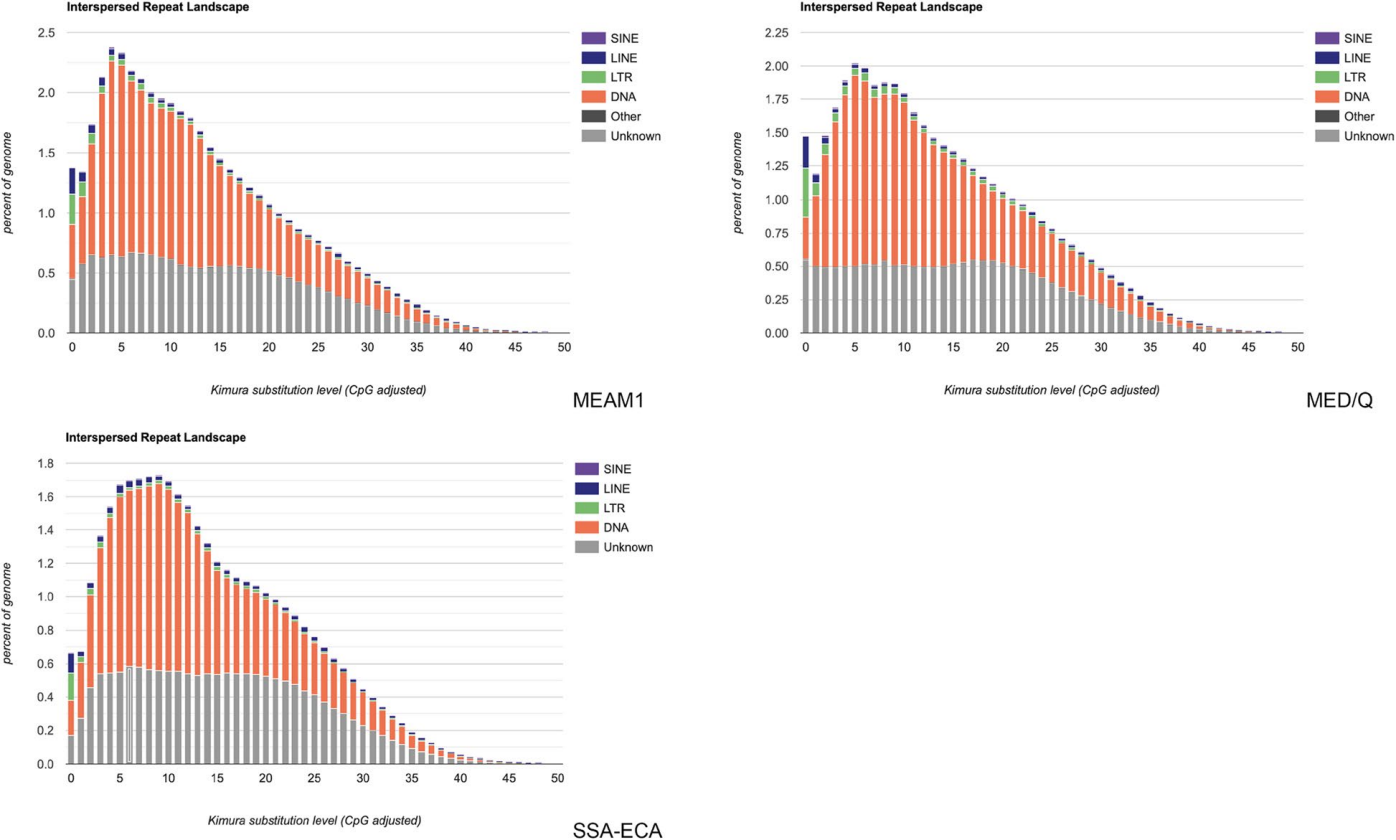
Repeat landscapes of the *B. tabaci* genomes. The repeat landscapes illustrate the activity of the different classes of transposable elements found in the three *B. tabaci* genomes. Sequence divergence scores were measured using Kimura distance which is represented on the x-axis while the percent coverage of the element in the genome is represented on the y-axis. Elements with low sequence divergence scores represent a more recent transposable element activity while elements with higher sequence divergence scores represent older transposition events

Figure 4 displays the repeat landscapes of the *B. tabaci* genome assemblies. SSA-ECA shows that the genome has one prolonged increase of TE activity around 5 to 10 Kimura score. The peak of activity can be found at 9 Kimura score having a genome proportion of 1.12% for DNA transposons, 0.02% for LTRs, 0.03% for LINEs, < 0.01 for SINEs, and 0.56% for unclassified TE sequences. MEAM1 has a peak of activity observed at 4 Kimura score having a genome proportion of 1.62% DNA transposons, 0.05% for LTRs, 0.06% for LINEs, 0.01 for SINEs, and 0.65% for unclassified TE sequences. MED/Q has a peak of activity at 5 Kimura score having a genome proportion of 1.43% for DNA transposons, 0.06% for LTRs, 0.03% for LINEs, 0.01% for SINEs, and 0.50% for unclassified TE sequences.

Further analysis of the superfamilies’ activities reveals that majority of the DNA transposon superfamilies peaked at around the same timeline as the peak of their activity in each of the whitefly genomes while retrotransposons’ peak of activity can be found at 0 Kimura score in all three landscapes (Supplementary Table 4).

## Discussion

This study is the first to characterize TEs found within the *B. tabaci* species complex and create a standardized annotation workflow that could be used to analyse future whitefly genome releases. The first three publicly available genomes of the species complex were the focus of this analysis (MEAM1, MED/Q, and SSA-ECA). Our results highlight that previously published data suggesting there are marked differences in TE classes between species [14–16] is due to erroneous identifications of TEs in the MED/Q draft genome. The improved and standardized TE annotation workflow developed will allow a more accurate analysis of the distribution of TE across the whitefly species complex in future studies.

### Identification of TEs in the genomes

The identification of TEs using the RepBase library yielded significantly lower results compared to the published results across the whitefly (Table 1) genomes while the RepBase library accurately identified TEs within the *D. melanogaster* genome (Table 2). In all three whitefly genomes, the TEs identified using the RepBase library were less than half of what was reported in their respective publications [14–16]. These results indicate that the RepBase library did not contain all the whitefly TE consensus sequences identified and published in respective previous studies [14–16]. Also as of April 12, 2019, RepBase is no longer publicly available and requires a subscription to access the up-to-date versions. These issues prevent further exploration of TEs within the species complex and have prompted the development of a TE annotation workflow that would standardize the annotation of multiple whitefly genomes.

The developed workflow was shown to accurately characterize TEs found within a genome using the *D. melanogaster* genome (Table 2). The repeats identified in the different *D. melanogaster* studies reported that < 20% of the genome is composed of TEs [51–54] and the results from the developed annotation workflow in this study were consistent with these findings. The similarities of the proportion of distribution of the TE orders shows the accuracy of the developed workflow. Research on *D. melanogaster* TEs date as far back as 1980 [59], and the TE annotation was able to identify these elements accurately.

This study attempted to run the TE annotation workflow described in the Chen et al. [14, 15] and Xie et al. [16] studies to compare results; however, the attempts did not yield similar results and some of the tools used failed to run with the other hemipteran genomes included in the study. The whitefly genome studies released their genome assemblies along with TE distribution content and GTF files for the TE copies; however, the TE consensus libraries were unavailable. These indicate that the TE libraries developed in the Chen et al. [14, 15] and Xie et al. [16] studies were not submitted to the RepBase library (or any other TE databases).

Within the whitefly genomes, the workflow developed was able to identify a similar proportion of TE orders within the MEAM1 and SSA-ECA genomes. Chen et al. [14, 15] reported the abundance of the DNA transposons in MEAM1 (29.25%) and SSA-ECA (25.94%) (Table 1). In contrast, in the MED/Q genome, LTRs were reported to be the most abundant element at 18.5%, with only 15.66% of the genome reported to be occupied by DNA transposons [16]. The results from this study show that the significant variation in the proportions of TEs found within these *B. tabaci* genomes is an artefact of the previous studies employing different TE annotation methods. In MEAM1 and SSA-ECA, a DNA transposon-specific identification tool was used while an LTR identification tool was included in the MED/Q annotation workflow. This study has highlighted the need for the implementation of a standardized workflow to accurately identify differences in TEs across genomes.

### TE content and genome assembly size

A positive correlation between TE content and genome size has previously been reported in arthropod genomes [34, 60, 61] as well as other genomes [18, 44, 62]. An arthropod wide study conducted by Petersen et al. [34] was the most extensive showing the association of genome assembly size and TE proportion within arthropod genomes. The largest genome included in the Petersen et al. [34] study (*Locusta migratoria* 5759.8 Mbp) has the largest TE proportion (63.55% genome proportion) whilst the smallest genome studied (*Belgica antarctica* 89.54 Mbp) has the lowest TE proportion (2.58% genome proportion).

The same positive correlation was identified across the six genomes included in this study. The *B. tabaci* genomes on average were larger than the non-whitefly genomes and contain more TEs (Fig. 2). The *M. persicae* assembly, the smallest non-whitefly genome included in the study (347.31 Mbp), had the lowest TE content (17.52%). Although TE content within genomes has been consistently shown to correlate with genome size [34, 60, 63], it remains unclear as to how exactly TEs directly contribute to this as different arthropod genomes have different landscapes of TEs. In lepidopterans, TE length and activity have been linked to genome expansion; however, the exact order(s) of TEs which contributed to the expansion remains unclear [61]. An association of a specific TE order (DNA transposons) and genome assembly size was identified in the *Clitarchus hookeri* genome [60]; however, the extent of the relationship has not yet been fully explored.

### TE classification

This study identified that the most abundant TE within the *B. tabaci* genomes are DNA transposons and are significantly higher within the whitefly species compared to the other hemipteran genomes included in the study. On average, the three whitefly genomes also had higher DNA transposons (22.85%) identified compared to the different arthropod clades that were analysed in the Petersen et al. study [34]; Hemiptera (3.24% average), Lepidoptera (1.40% average), Hymenoptera (2.83% average), and Drosophilids (1.67%).

DNA transposons are abundant in plant genomes and have been observed to have different roles; gene expression, genome expansion, gene regulation, and genome evolution [42, 60, 61]. DNA transposons can act as cis-regulatory elements which increase expression of nearby genes, and they can also decrease and silence gene expression because of small RNAs produced from them [41, 42].

In arthropods, DNA transposons have been observed to have a role in genome expansion [34, 60]. DNA transposons were identified to be the most abundant TE in the *C. hookeri* genome and comparison against the three other polyneopteran genomes shows an association of DNA transposons and genome assembly size [60]. The presence and absence of specific DNA TE superfamilies in the polyneopteran genomes have revealed the association; however, the mechanisms of the expansion due to the TEs require further exploration. The significant difference in DNA transposons found within the *B. tabaci* group could be one of the reasons why the genomes found in the species complex are larger in size compared to the other hemipteran genomes included in the study.

The abundance of DNA transposons within the species complex has been reported in MEAM1 [14] and SSA-ECA [15] genomes but was not explored further. The presence of common and unique DNA transposon superfamilies across the whitefly genomes highlights the importance of this TE order within the species. A more exhaustive exploration beyond characterization would be required to further understand the context of the presence of these elements within the species complex.

There are significantly fewer LINEs in the whitefly compared to the three non-whitefly genomes studied. On average, the three whitefly genomes also had less LINEs (1.05%) identified compared to the different arthropod clades analysed in the Petersen et al. study [34]; Hemipterans (5.14% average), Lepidoptera (5.17% average), and Drosophilids (4.34%). Most LINE studies in insects have been done on drosophilids. In *D. melanogaster*, strains that carried specific non-LTR retrotransposons exhibited hybrid dysgenesis [28, 64]. The progenies of these insects became sterile and had an increase in the frequency of mutations and chromosome rearrangement [28, 64]. LINEs have been observed to successfully maintain themselves through their host organism’s evolutionary lifetime [65–67]. Site-specific insertion of R1 and R2 LINE superfamilies near the 28S ribosomal RNA genes ensured its propagation while there is also evidence of another LINE superfamily successfully maintaining itself through non-site-specific insertion [65–68]. Different LINEs superfamilies can be found in the different insect genomes and each of these superfamilies could cause different effects depending on the type and the area of insertion [28, 34, 61, 69]. The consequences of the low distribution of LINEs within the whitefly species complex are unknown and an exploration of these elements in the wider context of insect evolution is warranted.

SINEs were the lowest identified TEs within the whitefly genomes. SINEs require LINEs for their transposition [28, 70] and the low distribution of SINEs within the species complex could correlate with the low distribution of LINEs. However, it should be noted that the workflow had difficulty identifying SINEs even when known SINEs were identified using the RepBase library (Table 1; Supplementary Table 1). The workflow was also unable to identify SINEs found within the *M. persicae* and *D. melanogaster* genomes.

The difficulty of identification of SINEs has been a consistent challenge in different arthropod TE studies. In the arthropod-wide TE identification performed by Petersen et al. [34], no SINE sequences were identified in seven of the 73 genomes included in the study. It is possible that there are genuinely no SINEs found within these genomes; however, there are multiple inconsistent reports of the proportions of identified SINEs found within the same organisms. Petersen et al. [34] reported 2.07% genome proport ion of SINEs within the *Heliconius melpomene* genome and 9.41% within the *B. mori* genome. In the *H. melpomene* TE analysis, Lavoie et al. [69] identified more at 8.22% genome proportion; whil e in the *B. mori* TE analysis done by Osanai-Futahashi et al. [71], 12.8% of the genome were identified as SINEs. The size of the retrotransposons adds to the difficulty of the identification of SINEs by automated TE annotation tools [72, 73]. SINEs being the shortest of the TEs would be impacted the most in the identification of these elements.

A significant percentage of TEs remain unclassified in the identified elements across genomes of the whitefly species complex. These unknown elements were screened for potential protein sequences and gene fragments; however, the screening yielded no positive results. Some of these unknown elements were also found to be shared across the three whitefly genomes. The significance of these unknown elements warrants further investigation and validation to enable improved classification and understanding of these elements.

Lastly, a third of the elements identified remain unknown, it should be noted that the distribution of classes amongst the TE class may change; however, DNA transposons in the *B. tabaci* species complex would remain the most abundant as more than half of the identified elements in the species complex are DNA transposons.

### The landscape of TEs and the superfamilies within the *B. tabaci* genomes

The repeat landscapes highlight the difference of the TEs and their activity across the *B. tabaci* genomes. The most abundant DNA transposon superfamily across the three whitefly genomes is the hAT superfamily. The hAT superfamily represents one of the most well characterised transposable elements and it also includes the first mobile DNA element that was discovered which was the Activator maize transposon [45, 74]. The hAT superfamily's general structure is 2.5-5 k bp with terminal inverted repeats that could span up to 50 bp, generating up to 8 bp of target site duplication (TSD) and encoding a single protein that includes a transposase domain [27]. There are 13 additional hAT superfamilies representing distinct lineages that appeared in this study. Some specific elements in the hAT family have been explored to identify their functions, structure, and evolution [27, 74]. hAT-related sequences are found in different organisms, including humans, nematodes, flies, fungi, and plants  [74].

For retrotransposons, there were three active repeat families found across the three *B. tabaci* genomes. Gypsy and Pao were found to be the most active LTR superfamily in all th ree genomes. Gypsy elements were first characterised in the *D. melanogaster* genome and their sequences have a high similarity with retroviruses of vertebrate animals [28]. Gypsy elements have high rates of transposition and are shown to insert themselves in introns and affect gene expression by disrupting normal transcriptional control [75, 76]. Pao elements are LTR elements that are related to the Gypsy element and are said to originate from the *Bombyx mori* genome [28, 77]. Pao elements encode a GAG and pol proteins and create a 4–6 TSD once they are inserted in the genome [25, 77]. In LINEs, RTE-BovB is the most active superfamily in the *B. tabaci* genomes. In the RepBase classification, BovB is classified under the RTE group where repeats in this group have the ability to encode their protein with two functioning domains; AP-endonuclease (Apurinic) and a reverse transcriptase [25, 27]. Bov-B (Bovine-B) elements have been identified in the *Bos taurus* genome and have been observed to have horizontal transfer events in other eukaryotic genomes [78–81].

The shape of the distribution of the repeats is similar within MEAM1 and MED/Q genomes. MEAM1 and MED/Q also share the greatest number of clusters. These two whitefly species are relatively closely related in phylogenetic analyses of the *B. tabaci* species complex [8, 82]. Aside from the shape of the distribution, the trends in the expansion are also similar between the two genomes as they both have the same superfamilies that are currently the most active, namely CACTA, hAT, RTE-BovB, Copia, Pao, and Gypsy. Copia’s activity was more prominent in the *B. tabaci* group with its presence being at low genome coverage in the non-whitefly group with the exception of the *A. pisum* genome assembly. Copia elements are autonomous LTR retrotransposons and their defining feature is the position of the integrase domain [27, 83]. Copia elements can be traced back further in plants while found to be more recently active in insects [84, 85]. They have been recently active in the *Drosophila* genome and it is hypothesized that they may be horizontally transmitted [85].

There is a decrease in the expansion of the activity of DNA transposon and an increase in LINE and LTR activity in the *B. tabaci* genome assemblies. It is still not fully clear how these trends affect their respective genomes. The relative age of the elements was identified using the Kimura substitution model; however, in order to place the element’s age within a more objective timescale, there is a need to determine the rate of evolution in whiteflies.

### Future of TE research in the whitefly species complex

With the availability of a standardized workflow and characterized TEs within the whitefly species complex, further investigation of the activity of these elements can now be performed. The impact that TEs have on biological properties (e.g., host plant colonisation, polyphagy, detoxification, virus transmission) and diversification of members of the whitefly species complex would be priority areas for further studies.

## Conclusion

TEs occupy a significant portion of whitefly genomes yet to date there have been no studies that characterise accurately the distribution of TEs found within the *B. tabaci* species complex. This study is the first to explore TE distribution within the species complex and to create a workflow to standardize the characterization of the elements across multiple whitefly genomes. The standardization of the TE annotation workflow has identified an abundance of DNA transposons within the species complex and has shown this to be true across all published *B. tabaci* genomes, contradicting previously published results [16]. Other TE superfamilies of note were also identified, some of these superfamilies were shown to be specific to the whitefly genomes. Unclassified elements remain significant, and the biological implications of the known elements also remain unknown. These issues highlight the need to explore further these elements within the different genomes of this whitefly species complex. The study has provided the groundwork for future TE studies within the species and hopes the initial characterization of these elements will increase interest in TEs found within the *B. tabaci* species complex.

## Methodology

### Genome data sets

Six different arthropod genomes were included in the study. Three of the genomes are from the *B. tabaci* cryptic species complex were included in the study; MEAM1 [14], MED/Q [16], and SSA-ECA [15]. The MEAM1 (*B. tabaci* Middle East-Asia Minor 1) genome assembly was obtained from GenBank under the accession number GCA_001854935.1. The MED/Q (*B. tabaci* Mediterranean) genome assembly was obtained from www.gigadb.org/dataset/100286. The SSA1-ECA (Sub-Saharan 1 East Central Africa) genome assembly was obtained from ftp://www.whiteflygenomics.org/pub/whitefly/SSA-ECA/v1.0/.

The three other arthropod genomes were non-whitefly genomes and were included to assess the performance of the workflow and compare the results of the TEs identified with the whitefly genomes; *Acyrthosiphon pisum* (project accession ABLF01000000)[86], *Diaphorina citri* (project accession AWGM01000000)[87], and *Myzus persicae* (project code LXJY01000000)[88]. All three genome assemblies were obtained from NCBI using their project accession codes.

### Repeat identification

The workflow performed in this study for creating a species-specific repeat library. The genome assembly to be studied is first submitted to MITETracker (https://github.com/INTABiotechMJ/MITE-Tracker) [89] and TransposonPSI_08222010 (http://transposonpsi.sourceforge.net/) for the initial step of the identification. The genome assembly was then submitted to genometools v1.5.9 (LTRHarvest and LTRDigest). Elements ranging from 100 to 6000 bps with terminal ending repeats with ≥ 85% similarity are identified as LTRs. The TEs representative sequences produced from MITETracker and genometools are then combined to create one library and is submitted to RepeatMasker to mask copies of the TEs found in the genome assembly. The masked genome assembly is then submitted to RepeatModeler v1.0.11 [90] for de novo TE identification. The masking of the copies of the already identified TE copies prevents RepeatModeler from identifying and modelling the repeat sequences that have already been identified. Utility scripts from the MAKER-P pipeline were also used to aid with the parsing of the results of genometools v1.5.9 (LTRHarvest and LTRDigest), RepeatModeler v1.0.11, and RepeatMasker v4.1.1 [91].

Each of the programs has candidate sequences that they have identified as repeat elements and the four outputs are subsequently merged into one library that is then submitted to USEARCH v11.0.667 [92, 93]. The clustering algorithm by USEARCH utilizes a algorithm called “greedy algorithm” which implements the “best” solution based on the current options. This means that sequence input order is important in the identification of candidate consensus sequences as the options for each cluster is based on the order of the sequences in the library. Sorting was performed using USEARCH’s “-sortbylength” command and the clusters were created based on ≥ 80% sequence similarity. A consensus sequence is then produced from each of the clusters to obtain a representative sequence. The representative sequences have ≥ 80% similarity to the member sequences. All the representative sequences have < 80% similarity to each other. The process reduces redundancy and assists in the identification of degenerated repeat elements.

The repeat library produced by the repeat identification workflow underwent several series of steps to classify each of the consensus sequences. The first method used for classification was the homology-based approach. The repeat library is submitted to RepeatClassifier(https://github.com/rmhubley/RepeatModeler/blob/master/RepeatClassifier) and the unclassified sequences were subsequently submitted to the web browser version of Censor [94]. Before continuing to the next step of the classification, sequences < 70 bp were removed and the sequences which were classified by the methods. The library was then submitted to TEClass v2.1.3 [95] and PASTEClassifier v1.0 [96]. Manual curation was done to analyse the results of both tools. The curation was based on sequence similarity and the length of the sequence aligned. The classification was accepted when both tools had similar results and spanned ≥ 80% of the element's length. When the results from the classification differ in the class level (i.e. DNA transposon and Retrotransposon), the element remained unknown. When the classification resulted in a difference in order (i.e. LINEs vs SINEs, LTR vs NonLTR) and ≥ 80% of the sequence length was identified, the element was classified using its more general level of classification. Any results that had less than 80% sequence length was disregarded..

A Blast search was performed on the unknown sequences against the NCBI nr protein database (version 2019.08.05) and UniProtKB/Swiss-Prot Arthropoda protein sequences obtained on July 10, 2019. The plan was to identify the unknown sequences with hits and parse through the results and remove the sequences with more than 50 bps hits from the species-specific library. None of the unknown sequences yielded a blast result and the unknown sequences were accepted as unclassified TEs.

Results from the homology-based classification, the consensus classification of TEClass and PASTEClassifier, and the unknown sequences were then combined to produce the final library. The process was repeated for each of the repeat libraries produced from the genomes included in this study.

### Genome assembly size and TE Distribution across species analysis

The proportion of TEs found within each genome were obtained from the RepeatMasker v4.1.1 output tables. The relationship between genome assembly size and TE content across the six genomes was tested using Spearman’s rank rho correlation. Spearman rank correlation tests the association between either two rank variables or one ranked and one measurement variable. The relationship identifies whether the variables covary (the variable increases/decreases when the other variable’s value changes).

A standard t-test and Wilcoxon rank-sum test were used to further compare the proportion of each order TEs across each group of genomes. Both tests compare the mean values of a measurement variable and identify if the mean values are significantly different. In this study, the tests identified whether there is a significant difference between the TE proportion of each order between the whitefly and non-whitefly genomes. The standard t-test was used for the TE orders with a similar variance while the Wilcoxon rank-sum test was used for values with non-normal distribution.

## Supplementary Information

**Additional file 1.** Supplementary Table 1.

**Additional file 2.** Supplementary Table 2.

**Additional file 3.** Supplementary Table 3.

**Additional file 4.** Supplementary Table 4.

## Data Availability

MEAM1, *A. pisum*, *D. citri*, and *M. persicae* genome assemblies are available at NCBI under the accession number GCA_001854935.1, project code ABLF01000000, project code AWGM01000000, and project code LXJY01000000.MED/Q genome assembly is available at www.gigadb.org/dataset/100286. SSA1-ECA genome assembly is available at ftp://www.whiteflygenomics.org/pub/whitefly/SSA-ECA/v1.0/. The species–specific repeat libraries have been submitted to DFAM and is currently under review. The species-specific repeat libraries and the associated downstream analysis script are also available from the corresponding author upon request.

## Abbreviations

BAC: Bacterial Artificial Chromosome
GTF: Gene transfer format
LINE: Long interspersed nuclear elements
LTR: Long terminal repeat
MITE: Miniature inverted repeat transposable element
NonLTR: Non-long terminal repeat
SINE: Short interspersed nuclear elements
TE: Transposable element
