# Correction: Characterization of transposable elements within the *Bemisia tabaci* species complex

**DOI:** 10.1186/s13100-022-00273-3

**Published:** 2022-06-08

**Authors:** Juan Paolo A. Sicat, Paul Visendi, Steven O. Sewe, Sophie Bouvaine, Susan E. Seal

**Affiliations:** 1 Natural Resources Institute, University of Greenwich, Central Avenue, Gillingham, Chatham, ME4 4TB UK; 2 Centre for Agriculture and the Bioeconomy, Queensland University of Technology, Brisbane, QLD 4000 Australia

**Correction: Mobile DNA 13, 12 (2022)**

**https://doi.org/10.1186/s13100-022-00270-6**

Following the publication of the original article [1] the authors requested to change the wording in Acknowledgements and Funding sections. The original article [1] has been updated.

The revised wording should be:
